# Patterns of healing: a comparison of two proximal tibial osteotomy techniques

**DOI:** 10.1007/s11751-016-0243-9

**Published:** 2016-02-16

**Authors:** Anna C. Peek, Anna Timms, Kuen F. Chin, Peter Calder, David Goodier

**Affiliations:** Royal National Orthopaedic Hospital, Stanmore, UK

**Keywords:** Regenerate, Osteotomy, Healing

## Abstract

Several low-energy osteotomy techniques are described in the literature, but there is limited evidence comparing them. Our study evaluates the patterns of regenerate formation using two different osteotomy techniques. Two cohorts of patients underwent osteotomy of the tibia using a Gigli saw (*n* = 15) or De Bastiani corticotomy (*n* = 12) technique. The patient radiographs were assessed by the two senior authors who were blinded to the osteotomy type. Regenerate quality was assessed along the anterior, posterior, medial and lateral cortices, graded 1–5 from absent to full consolidation over time. The time to 3 cortices healed/regenerate length was calculated. The time to consolidation of the anterior, posterior, medial and lateral cortices was compared. The mean 3 cortices index in the Gigli group was 2.0 months/cm and in the De Bastiani group 1.8 months/cm. This was not a significant difference. In both groups, anterior bone formation was slower, and anterior cortical deficiency with a scalloped appearance was seen in 25 % of cases overall with no statistically significant difference between the two groups. Both Gigli saw and De Bastiani corticotomy techniques result in good bone formation following distraction osteogenesis. The anterior tibial cortex consolidates more slowly than the other cortices in both groups. This is likely due to deficient soft tissue cover and direct periosteal damage at time of osteotomy.

## Introduction

Limb lengthening and segmental bone transport are techniques based upon the principles of ‘Callus Distraction Osteogenesis’ discovered by Ilizarov in the 1950s. Ilizarov pioneered the use of a low impact division of the cortex of bone, attempting to preserve the medullary blood supply (‘corticotomy’) followed by a latent period of 5–7 days to allow callus to start forming, then gradual distraction in increments totalling 1 mm per day [1].

This corticotomy technique has been adapted by various authors, notably De Bastiani [2] and Patkiss and Gross [3] who described the ‘Afghan Percutaneous Osteotomy’ using a Gigli saw. The De Bastiani technique purports to keep the medullary blood supply intact; the Gigli saw technique divides it. On the other hand, the usual approach for the De Bastiani corticotomy is an anterior incision over the tibial crest, whereas the incisions to pass a Gigli saw tend to be smaller but require more periosteal elevation.

We had observed in our limb reconstruction practice that the subcutaneous border of the tibia (shown on AP and lateral radiographs as the most anterior cortex on lateral projection and most medial cortex on AP) was the slowest to ‘fill in’ after corticotomy. Our study was to evaluate whether there was a difference in formation of bone in this area between these two techniques.

## Method

We conducted a retrospective review of patients undergoing limb reconstruction surgery identified using a prospectively collected database. Indications for surgery included tibial lengthening or bone transport for defect reconstruction with a minimal distraction of 2 cm (Table 1). There was no significant difference in indication between the 2 groups (Chi-square, p value NS). Children and patients with metabolic bone disorders were excluded. The osteotomy for bone distraction was performed in the proximal tibia with a Gigli saw in 15 patients (GS group) and by the De Bastiani technique in 12 patients (DB group). The mean age was similar in both groups (DB 36 years, GS 41 years *p* value NS).

**Table 1:** Indications for frame, number of patients

Group	Lengthening or deformity correction	Aseptic nonunion or malunion	Infected nonunion
GS	2	11	2
DB	6	5	1

## Surgical technique [4]

- Gigli saw
  Two transverse incisions are made and via subperiosteal dissection a suture is passed from the posteromedial to anterolateral using a right-angled and curved clamp. The Gigli saw is tied to the suture and is pulled from posterior to anterior. Elevators are inserted, and the posterior and lateral cortices divided. The medial periosteum is then elevated, and the cortex divided. The saw is cut with a wire cutter and removed.

- De Bastiani
  A small 1-cm incision is made over the anterolateral aspect of the tibia. The periosteum is elevated along the anterior and lateral cortices. The tibia is predrilled from anterior to posteromedial and posterolateral, 3–5 drill holes. An osteotome is then passed along the anterior and lateral cortices. The osteotomy is completed by rotation of the osteotome.

The osteotomy technique was based on the surgeon’s choice. Following osteotomy, a latent period of 6 days was followed by lengthening of 1 mm per day in four quarterly turns with Ilizarov frames and 1 mm per day in a single correction using Taylor Spatial Frames. The type of frame was also the surgeon’s choice. Follow-up including radiographs was every 2 weeks during the lengthening period and every 4–6 weeks during regenerate consolidation.

The patient radiographs during lengthening and consolidation periods were anonymised, and the regenerate assessed by the two senior authors, in an attempt to blind the assessors to the type of osteotomy performed.

**Fig. 1:**
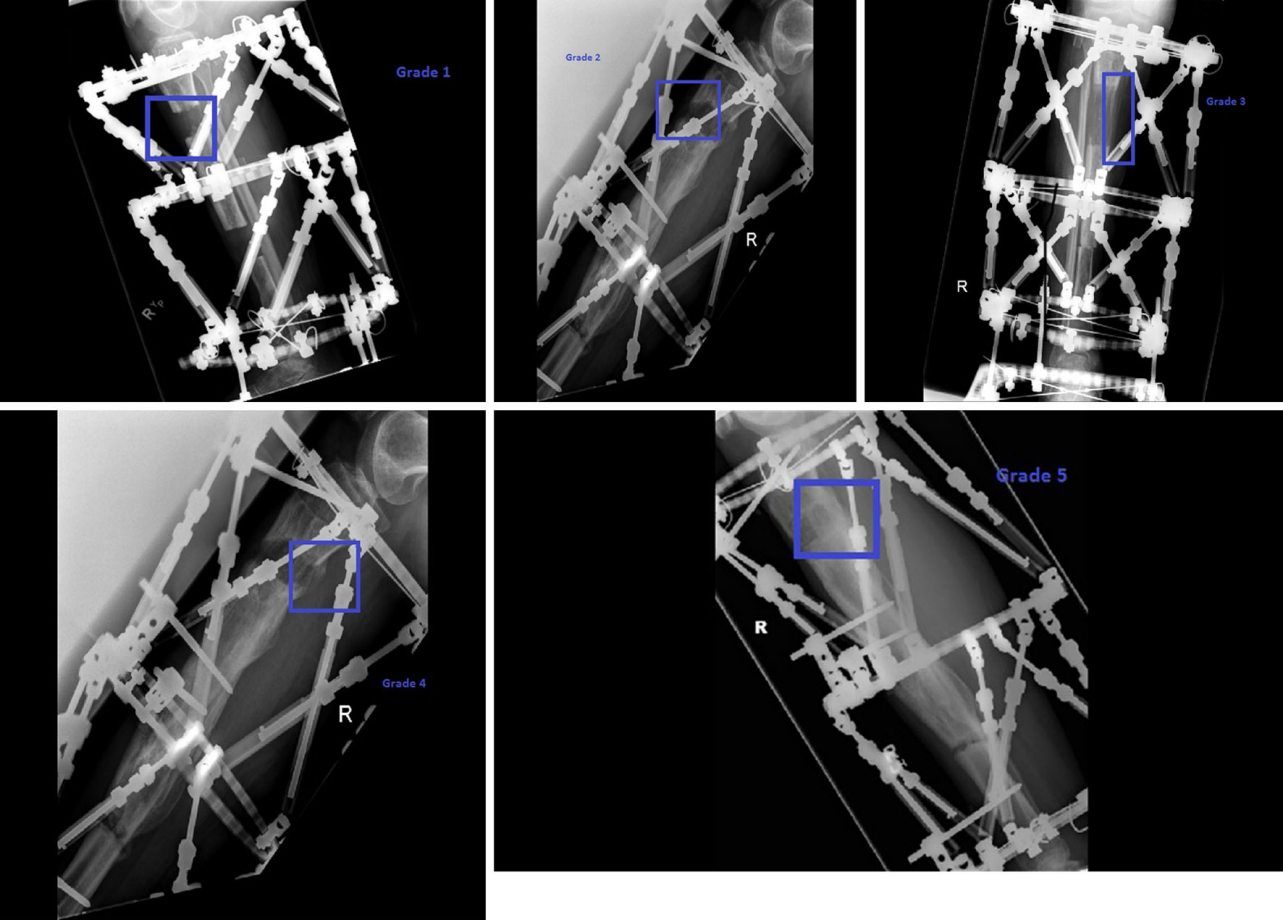
Illustrations of the scoring system

The bone quality of the regenerate was recorded along the anterior, posterior, medial and lateral cortices. This was graded 1–5, from absent to full consolidation over time in frame. Each cortex was graded independently (Table 2; Fig. 1).

**Table 2:** Cortex grading

Grade of cortex	Appearance of cortex
1	No visible callus
2	Concave callus
3	Straight callus
4	Convex callus
5	Consolidated

A modified healing index was used as the time for a minimum of 3 cortices to consolidate [5]. This measure was used rather than the healing index to frame removal, as some patients spent a considerable length of time in frame undergoing bifocal treatment. The proximal regenerate had consolidated, but there was considerable delay in waiting for the transport docking site to unite.

The time to consolidation of the anterior, posterior, medial and lateral cortices was compared between the two osteotomy techniques.

Statistical analysis was performed using SPSS software version 22. The Chi-square test and paired T test were used, and significance level was set at *p* < 0.05.

## Results

The overall time to 3 cortices grade 4 was similar: 2.2 months per cm in the GS group and 1.8 months per cm in the DB group (Chi-square, *p* value NS).

**Fig. 2:**
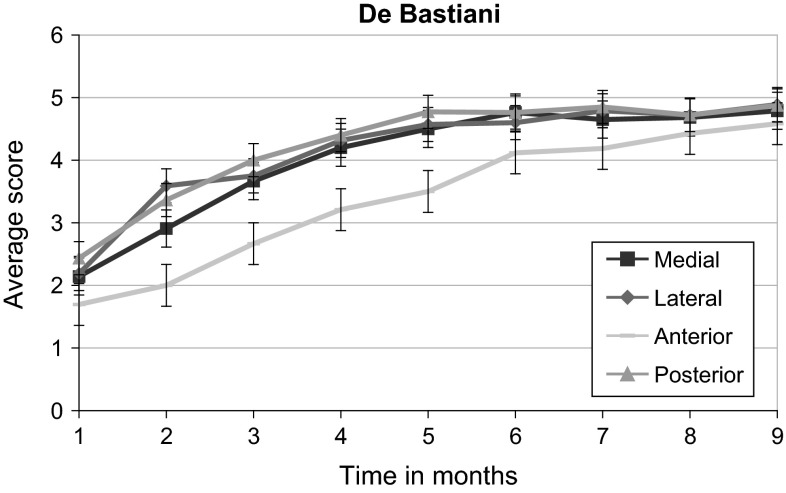
Average score of each cortex vs time since corticotomy in the De Bastiani group

**Fig. 3:**
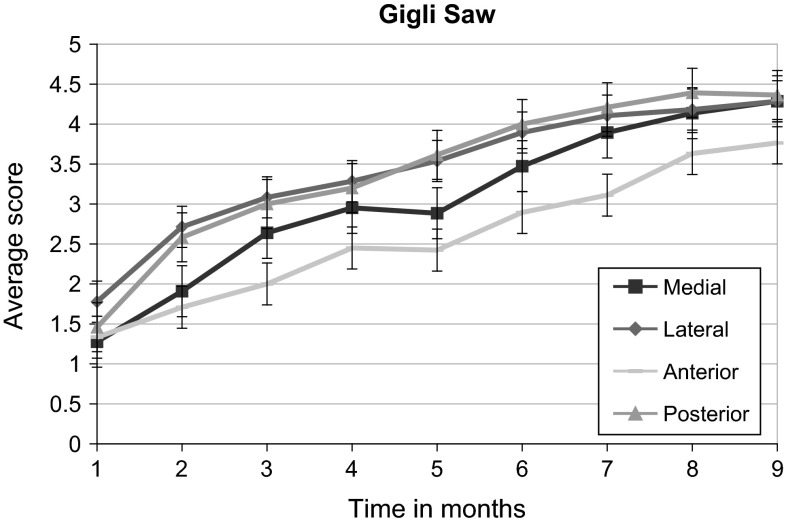
Average score of each cortex vs time since corticotomy in the Gigli saw group

The anterior cortex was slower to heal than the posterior (Chi-square, *p* < 0.05) and lateral cortices in both groups (Chi-square, *p* < 0.05); although there was no statistically significant difference between the two groups, the trend seemed more marked in the anterior cortex relative to the other cortices in the DB group (Figs. 2, 3). Although the absolute scores were lower in the GS group in comparison with the DB group, this was not statistically significant (paired *t* test, *p* value NS).

Anterior cortical deficiency with a scalloped appearance was seen in 25 % of cases overall at some point during treatment, with no statistically significant difference between the two groups (13 % GS vs. 41 % DB, Chi-square *p* = 0.09).

## Discussion

Experimental and clinical studies examining the factors influencing regenerate formation have been described [6, 7]. These include the site, the age of the patient and the amount of distraction applied. In a canine model, tibial osteotomies made with a mallet and hammer, drill holes and an osteotome, or an oscillating saw were compared. In this study, fewer bone divisions made using the oscillating saw were consolidated at 10 weeks [8]. In a direct comparison in 41 patients between a Gigli saw Afghan osteotomy and an osteotomy made with drill holes and an osteotome, the Gigli saw method was found to result in a shorter healing index [9]. The authors concluded the Gigli saw-type osteotomy caused less periosteal disruption.

We note proximal tibial osteotomies demonstrate a distinct pattern of healing with the anterior cortex in particular lagging behind the other cortices. Although it is more apparent in the De Bastiani group, there is no statistically demonstrable difference between the two, concluding that this pattern of healing relates primarily to the soft tissue attachments in this region of the tibia rather than any periosteal striping or thermal damage provoked at the time of the osteotomy. One might assume that, due to the triangular shape of the tibia at this level, posterior healing conveys more stability to the regenerate, but this was not tested in this study.

We have not demonstrated any significant difference in the healing index between the two methods in this study.

The limitations of this study are its retrospective nature and the small numbers studied with a potential for a type 2 error. Additionally, use of an unvalidated scoring system for the grading of the cortical appearance will introduce bias. The two groups were treated by different surgeons, and there may be unknown confounding factors related to this.
